# Analysis of the correlation between periodontal disease and metabolic syndrome among coal mine workers: A clinical study

**DOI:** 10.1097/MD.0000000000021566

**Published:** 2020-08-14

**Authors:** Jian Zhao, Xiang-yu Zhu, Yan Ren, Jin-yuan Li

**Affiliations:** a Kailuan General Hospital, Tangshan; b School of stomatology, North China University of Science and Technology, Hebei China.

**Keywords:** coal mine workers, clinical trials, periodontal disease, metabolic syndrome

## Abstract

Metabolic syndrome (MetS) refers to the pathological state of metabolic disorders in the body's proteins, fats, carbohydrates and other substances. MetS is a systemic metabolic disease. Periodontal disease is also a part of systemic inflammatory diseases. Among Chinese patients with middle-aged and elderly MetS, the periodontal morbidity is very high, which is due to the involvement of inflammatory mediators in the pathogenesis of MetS and periodontal disease. The latter may also be a risk factor for the former's morbidity and promotion of disease progression. At present, there are not many investigations and studies on periodontal examination data and periodontal disease prevalence of patients with MetS. Coal mine workers, especially coal mine underground workers, have different work natures and different working environments. See related report.

We will collect the clinical diagnosis and treatment information of the enrolled patients. We will focus on checking the incidence of periodontal disease and recording. Establish a database, check every 10 medical records, and make corrections in time to ensure data accuracy. We will popularize oral hygiene knowledge for the included patients and guide them to brush their teeth correctly and how to use dental floss. We will perform periodontal examination on the patients’ teeth by site and record the plaque index, gingival sulcus bleeding index, periodontal pocket exploration depth and other indicators. We will repeat the above inspection items and record in the second and fourth weeks of the experiment.

This study will explore the correlation between periodontal disease and MetS of coal mine workers. We aim to clarify the role and mechanism of MetS in the occurrence and development of periodontal diseases, guide the prevention of periodontal diseases, and thus reduce the prevalence of periodontal diseases.

**Trial registration:**

ClinicalTrials.gov, ChiCTR2000034177, Registered on 27 June 2020

## 1 Introduction

Periodontal disease refers to diseases that occur in periodontal tissue, including gum disease that only affects gum tissue and periodontitis that affects deep periodontal tissue (peridontal membrane, alveolar bone, cementum).^[1,2]^ Periodontal disease is the second largest oral disease after caries, and it is also an important disease that harms human oral health.^[3]^ About 2-thirds of people in China suffer from periodontal disease and are related to a variety of diseases. The early symptoms of periodontal disease are not easy to attract attention, resulting in chronic infection of periodontal tissue and repeated attacks of inflammation.^[4]^ This not only impairs the function of the oral chewing system, but also seriously affects health. Various factors can trigger or promote the occurrence and development of periodontal disease, and become a risk factor for periodontal disease.^[5]^ Metabolic syndrome (MetS) refers to the pathological state of metabolic disorders in the body's proteins, fats, carbohydrates and other substances. MetS is a systemic metabolic disease.^[6]^ It can cause disorder of the internal environment of the body and uncontrolled inflammation, which can be reflected in various organs of the body and cause various diseases.^[7]^ MetS is associated with many chronic diseases. The core of its pathogenesis is obesity and insulin resistance, and it is accompanied by an imbalance of inflammatory mediators and a low-level inflammatory response, leading to multiple systemic diseases in the body.^[8,9]^ The disease has the following characteristics: ① A variety of metabolic disorders are integrated, including obesity, high blood sugar, hypertension, dyslipidemia, high blood viscosity, high uric acid, high incidence of fatty liver and hyperinsulinemia. These metabolic disorders are the pathological basis of heart and cerebrovascular diseases and diabetes. It can be seen that diabetes is not an isolated disease, but 1 of the components of the MetS. ② There is a common pathological basis, and it is currently believed that their common cause is insulin resistance and hyperinsulinemia caused by obesity, especially central obesity. ③ MetS can cause a variety of diseases, such as hypertension, coronary heart disease, stroke, and even certain cancers, including breast cancer related to sex hormones, endometrial cancer, prostate cancer, pancreatic cancer of the digestive system, liver and gallbladder cancer, Colon cancer, and so on.

Periodontal diseases are also part of systemic inflammatory diseases. Among Chinese middle-aged and elderly patients with MetS, periodontal morbidity is very high. This is due to the involvement of inflammatory mediators in the pathogenesis of MetS and periodontal disease. The latter may also be a risk factor for the former's morbidity and promotion of disease progression. At present, there are not many investigations involving periodontal examination data and periodontal disease prevalence of patients with MetS. However, there are no domestic reports on coal mine workers, especially those working underground. This study will explore the relationship between MetS (including management personnel, uphole workers, and downhole workers) and explore the significance of MetS in the occurrence and development of periodontal disease, and investigate and analyze in different positions and different jobs Differences in morbidity in the environment. We will hope to provide a reference for the prevention and treatment of periodontal diseases.

## 2 Methods/design

### 2.1 Study design and settings

**Figure 1:**
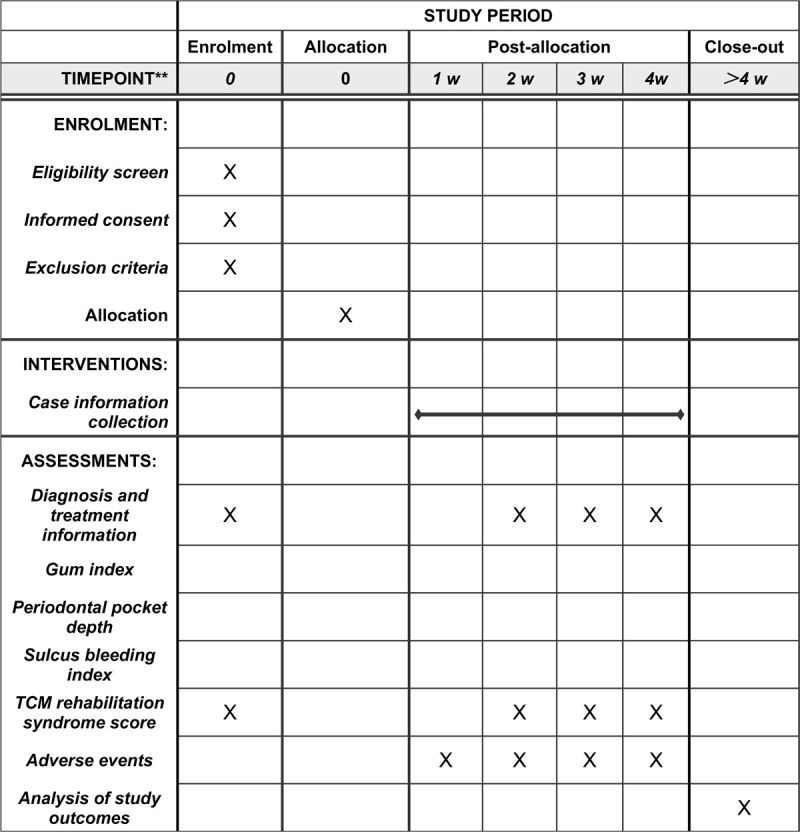
SPIRIT figure for the schedule of enrollment, interventions, and assessments.

This study will be conducted at the Kailuan general hospital (Tangshan City, Hebei Province). Researchers in this subject have long been engaged in clinical diagnosis and treatment and clinical teaching of periodontal diseases. We have a wealth of theoretical knowledge and operational experience, and master the cutting-edge development of various diagnosis and treatment technologies at home and abroad. This protocol was written and based on Standard Protocol Items: Recommendations for Interventional Trials guidelines The participants will be informed about the research, procedures, risks, and benefits by JZ (author of this protocol). If they agree, they will sign an informed consent form. Only those participants who read and agree to the protocol and who sign the informed consent form will take part of the study, following the schedule described in Figure 1.

### 2.2 Participants

The subjects of this study will be included in the outpatient and hospitalized patients from the Kailuan general hospital, and meet the diagnostic criteria for MetS.

#### 2.2.1 Diagnostic criteria

We will refer to the relevant diagnostic criteria recommended by the Diabetes Branch of the Chinese Medical Association:

(1) Overweight and/or obese BMI≥25.
(2) Hyperglycemia fasting blood glucose (FPG) ≥6.1mmol/L (110 mg/dL) and/or 2hPG ≥7.8mmol/L (140 mg/dL), and/or those who have been diagnosed with diabetes and treated.
(3) Hypertensive systolic/diastolic blood pressure ≥140/90mm Hg, and/or those who have been diagnosed with hypertension and treated.
(4) Blood lipid disorder fasting blood triglyceride ≥1.7mmol/L (150 mg/dL), and/or fasting blood HDL-C <0.9mmol/L (35 mg/dl) (male), <1.0mmol/L (39 mg/dL) (female).

Those with 3 or all of the above 4 components can be diagnosed with MetS.

#### 2.2.2 Inclusion criteria

This study will be conducted in China. Patients will be recruited from Plastic surgery departments of the Kailuan general hospital. We will enroll participants based on the following inclusion criteria:

(1) Meet the diagnostic criteria of Western Medicine MetS.
(2) Occupation is coal mine employees (including management personnel, underground workers, underground workers).
(3) The age is between 20 to 55 years old.
(4) Those who have voluntarily signed the informed consent;

#### 2.2.3 Exclusion criteria

Patients will be excluded if they meet the following criteria:

(1) Patients with severe diseases such as malignant hypertension, severe arrhythmia, acute myocardial infarction, cerebrovascular accident, and so on.
(2) Patients with acute infection, trauma, surgery, diabetic ketoacidosis, and hyperosmolar coma recently.
(3) Patients with severe primary diseases such as brain, liver, kidney and hematopoietic system, mental illness and cognitive dysfunction.
(4) Patients with tumor and other organ failure;

#### 2.2.4 Conditions for participants to suspend and withdraw from the clinical trial

Researchers participating in clinical trials should carefully record the reasons for the suspension of the trial and the relationship with the clinical trial. It is necessary to clearly record the unwillingness of the subjects to continue the clinical trials, put forward the reasons for withdrawing from the clinical trials, and record the evaluation indicators at the time of discontinuation in detail.

(1) The main symptoms are not clear; the extended description of the current medical history is incomplete; the chief complaint or the description of the current medical history does not match the diagnosis of this disease.
(2) Incomplete records of tongue coating and pulse of traditional Chinese medicine affect the syndrome judgment of this disease.
(3) The lack of or incomplete general information and physical and chemical index information affects the analysis of relevant data.

### 2.3 Interventions

We will collect the clinical diagnosis and treatment information of the enrolled patients (such as symptoms, syndromes, signs, electrocardiogram and laboratory examination data, physical examination data, and so on). We will focus on checking the incidence of periodontal disease and recording. Establish a database, check every 10 medical records, and make corrections in time to ensure data accuracy. First of all, we will popularize oral hygiene knowledge for the included patients and guide them to brush their teeth correctly and use dental floss. We will perform periodontal examination on the patients’ teeth by site and record the plaque index, gingival sulcus bleeding index periodontal pocket exploration depth and other indicators. After the inspection, the periodontal pockets were rinsed with 3% hydrogen peroxide solution and 0.9% sterile saline. We will repeat the above inspection items and record in the second and fourth weeks of the experiment. None of the patients in the 2 groups received systemic antimicrobial therapy, and the above clinical operations were performed by the same doctor.

### 2.4 Statistical analysis.

(1) Collect clinical diagnosis and treatment information (such as symptoms, signs, electrocardiogram, tongue and laboratory examination data, physical examination data, and so on), establish a database, verify every 10 medical records entered, and correct in time to ensure Data accuracy.
(2) The data is analyzed with the help of SPSS25.0 statistical software. The measurement data is expressed by x ± s or M (IQR), the count data is expressed by the composition ratio or rate, and the chi-square test is used for the comparison between the groups. The model analyzes the relationship between relevant syndromes and clinical indicators.

### 2.5 Data management

**Figure 2:**
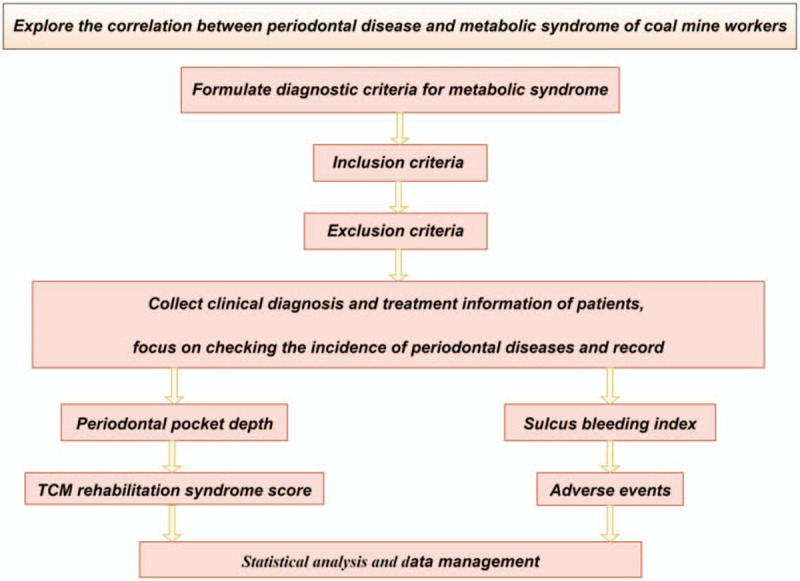
Study design flow chart.

Data management uses EXCEL software to build a database, double entry, check for outstanding values, and lock. Information obtained from the evaluation of each participant will be recorded on a paper print-out. The information will then be handwritten on a paper document case report form and entered into an Excel file for future statistical analyses. In accordance with the Personal Information Protection Act, the names of all participants will not be disclosed, and a unique identifier number given during the trial will be used to identify participants. All of the participants will be informed that the clinical data obtained in the trial will be stored in a computer and will be handled with confidentiality. The participants’ written consent will be stored by the principal investigator. (Fig. 2)

### 2.6 Ethics

The study will be conducted under the Declaration of Helsinki principles, as well as following the norms of good clinical practice. Recruitment of patients has not started in this study. The study plan will be submitted to the ethics committee of the Kailuan General Hospital for review. The study protocol will be approved by the ethics committee of Kailuan General Hospital. The protocol of this study has been registered in the Chinese Clinical Trial Registry with the number ChiCTR2000034177.

## 3 Discussion

China is a country with a high incidence of periodontal disease, 80% to 90% of adults suffer from varying degrees of periodontal disease.^[10]^ According to the results of the third national oral health epidemiological survey in 2000, the number of patients with periodontitis in urban and rural areas in China is as high as hundreds of millions.^[11]^ However, due to poor health awareness and inadequate prevention and early treatment, periodontal diseases have seriously affected the oral health of the Chinese people, and even overall health.^[12,13]^ With the improvement of living standards and lifestyle changes, the incidence of MetS in our country has increased significantly. The prevalence of MetS in the population reaches 25%.^[14,15]^ As an independent risk factor for various diseases, MetS has begun to attract people's attention for its role in the occurrence and development of periodontal diseases. The cause of MetS is not yet clear. It is currently believed to be the result of the interaction of multiple genes and multiple environments, and is closely related to genetics and immunity.^[16]^ The disease is affected by a variety of environmental factors, and is concentrated in a high-fat, high-carbohydrate diet structure, increasing the occurrence of insulin resistance, low labor intensity, and low physical activity resulting in the occurrence and development of MetS.^[17]^ The main clinical manifestations of MetS are abdominal obesity or overweight, abnormal lipid metabolism, hypertension, diabetes, insulin resistance and/or abnormal glucose tolerance.^[18]^ The main clinical manifestations of periodontal disease are gingival inflammation, bleeding, periodontal pocket formation, alveolar bone resorption, alveolar bone height reduction, tooth loosening, displacement, and weak chewing. In severe cases, the teeth may fall off or cause tooth extraction.^[19]^ May have complications such as pain, pus, and bad breath. Local complications of periodontal disease include periodontal abscesses and loose teeth. Systemic effects are generally small.^[20]^ Some scholars believe that it may be related to certain rheumatic diseases. Chronic inflammation develops gradually with repeated episodes. Clinically, it is mainly characterized by alveolar bone absorption and loosening of teeth. It gradually causes occlusal trauma and displaces the teeth.^[21]^ Finally, it causes missing teeth and poor support of the remaining teeth, resulting in difficulties in repair and treatment. At present, there are multiple classification methods for periodontal disease, but it is mainly divided into gingivitis, periodontitis, periodontal trauma, juvenile periodontitis and periodontal atrophy.^[22]^ Gingivitis is mainly limited to inflammatory lesions of the gum tissue, generally does not involve deep periodontal tissue. Periodontitis is most common in periodontal disease, mainly including gingival redness and bleeding, periodontal pocket formation, periodontal pocket overflowing pus, loose teeth, gingival recession, periodontal abscess and so on.^[23]^ Periodontitis is mainly characterized by the formation of pathological periodontal pockets. Periodontal trauma is a disease in which periodontal support tissue is damaged due to excessive bite pressure or abnormal direction, which exceeds the load that the periodontal tissue can bear. It develops slowly and generally has no obvious symptoms. Sometimes it feels weak to chew, or sometimes there is hidden or dull pain.^[24]^ Juvenile periodontitis is a chronic degeneration of the periodontal tissue that affects most teeth. It is characterized by the fact that most of the patients are younger and the lesions develop rapidly, so that the teeth appear loose, displaced, and periodontal pockets are formed at the early stage of the disease, and then secondary infection. Pathogenic factors may be related to heredity. Periodontal atrophy is mainly a regressive lesion of the gum and alveolar bone tissue. Manifested as gingival recession and exposed neck or root. The main causes of periodontal atrophy are: compression of the gums of the tooth neck on the gums; long-term disuse of the teeth at the site or systemic factors; mechanical stimulation caused by incorrect brushing methods; compression of the gums by the restoration.

This study will explore the correlation between periodontal disease and MetS of coal mine workers. We aim to clarify the role and mechanism of MetS in the occurrence and development of periodontal diseases, guide the prevention of periodontal diseases, and thus reduce the prevalence of periodontal diseases. In addition, this study will help to control the development of periodontal diseases, improve oral health and general health, and reduce the consumption of medical resources caused by periodontal diseases.

## Acknowledgments

The authors would like to thank all the trial participants. The authors are grateful for the support for this study: trial coordinating team, surgical staff, nurses, and research departments.

## Author contributions

JZ and YR designed the study protocol and drafted the manuscript. XYZ reviewed the study protocol and drafted the manuscript. JYL is responsible for the statistical design and analysis as trial statistician. All authors carefully read and approved the final version of the manuscript.
